# Improving the management of Insomnia in Community General Adult Mental Health Care

**DOI:** 10.1192/j.eurpsy.2026.11991

**Published:** 2026-07-31

**Authors:** J. G. Heslop

**Affiliations:** South West London and St George’s Mental Health NHS Trust, London, United Kingdom

## Abstract

**Introduction:**

Sleep disturbance is a prevalent issue, affecting approximately 78% of individuals with mental health disorders. Evidence-based treatments such as cognitive behavioural therapy for insomnia (CBT-i), sleep hygiene practices, and hypnotic medications are known to be effective. This quality improvement project aimed to assess current clinical practice and enhance the management of insomnia in community general adult psychiatric care, aligning with national guidelines (Donaldson et al., BJMP 2009; NICE CKS, 2025).

**Objectives:**

The primary aim was to improve the recognition and management of insomnia. A secondary aim was to develop and implement educational and procedural interventions that promote adherence to national guidance. A closed-loop audit cycle was used to evaluate the prevalence of insomnia and assess the impact of targeted interventions on clinical practice.

**Methods:**

An initial audit cycle reviewed clinical records from 48 randomly selected patients seen over the previous five months. Data were collected on the identification and management of insomnia, namely the use of sleep hygiene advice, CBT-i techniques, and hypnotic prescribing. A multidisciplinary focus group informed an education programme which was delivered to the clinical team, covering sleep physiology, national treatment guidelines, and practical CBT-i principles (e.g. sleep restriction and cognitive restructuring). A simplified sleep management algorithm was developed and distributed (see image 2). Following these interventions, a second audit cycle was completed.

**Results:**

1) Insomnia prevalence was 58% and 63& in audit cycle 1 and 2 respectively (see all data in Table 1).

2) Sleep hygiene advice was given to 36% and 45% of relevant patients in audit cycle 1 and 2 respectively.

3) CBT-i strategies were used in 11% of cases in audit cycle 1, and increased to 27% in audit cycle 2.

4) Hypnotic prescribing remained limited. Zopiclone and promethazine were the most used. Combination hypnotic use was observed in 15% and 8% of patients in audit cycle 1 and 2 respectively.

**Image 1:**
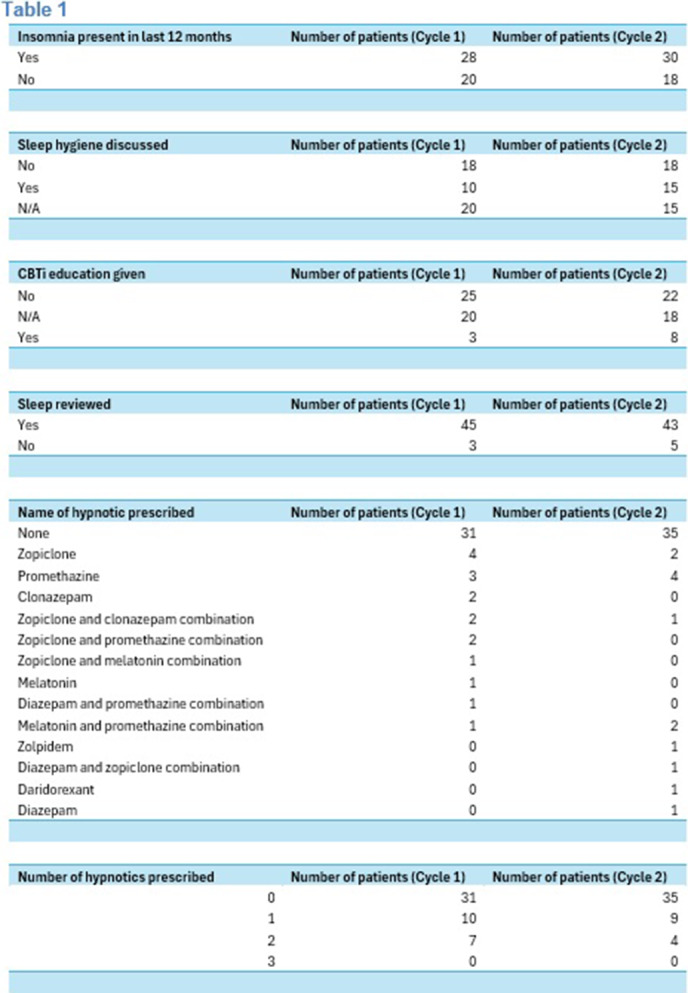
Image 1: Long description.

**Image 2:**
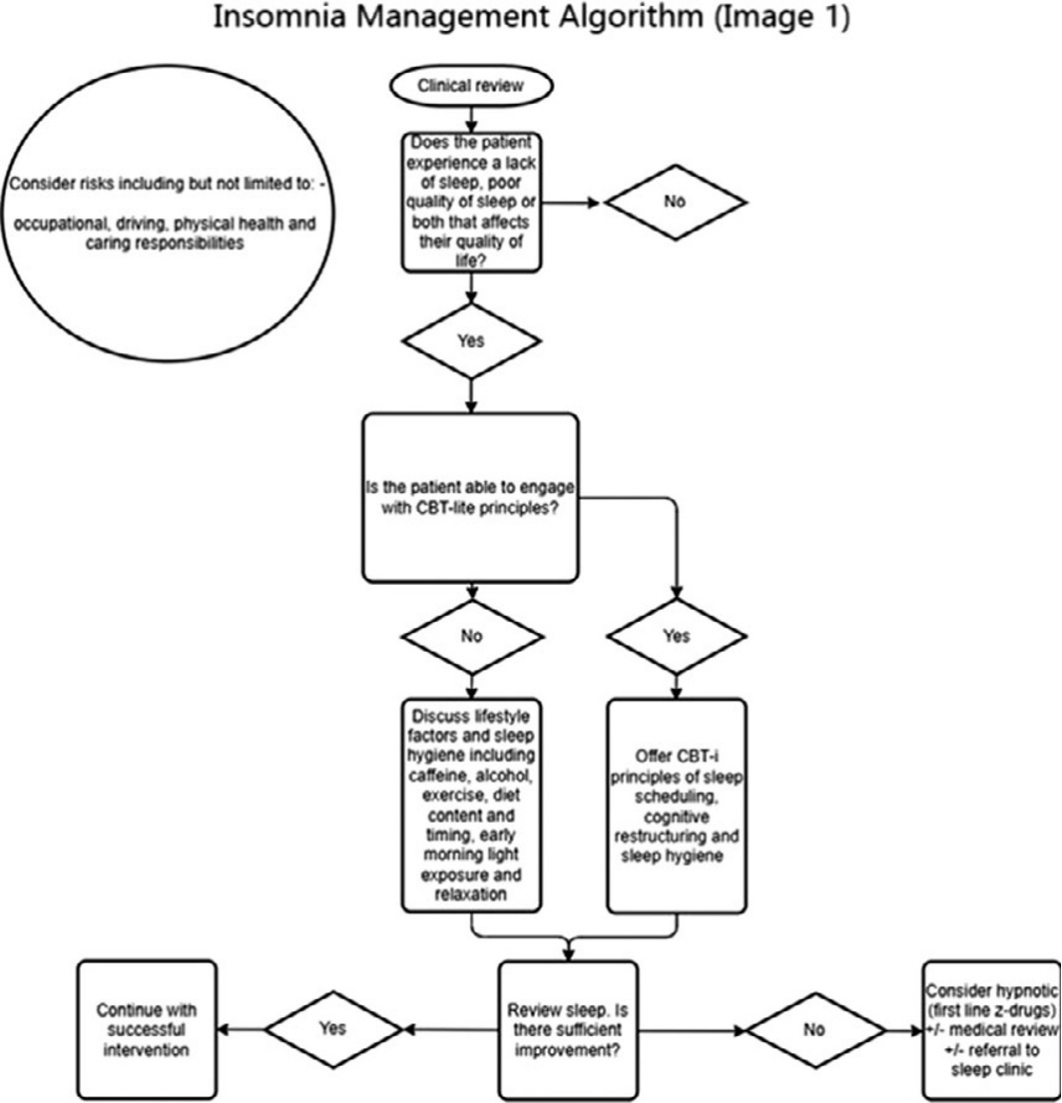
Image 2: Long description.

**Conclusions:**

1) Insomnia is common in community psychiatric practice but is often under-managed.

2) Modest improvements in the delivery of sleep hygiene and CBT-i interventions were seen following the project.

3) Sleep hygiene remains the most commonly used intervention, likely due to greater clinician familiarity and lower perceived complexity.

4) Despite being first-line treatment, CBT-i remains underutilised—potentially due to limited training, time constraints, and low clinician confidence.

5) Positive change is achievable in high-acuity settings, though sustained effort and system-level support are required.

**Disclosure of Interest:**

None Declared

## Long descriptions

### Image 1: Long description

The table is divided into six sections, each with columns for Number of patients Cycle 1 and Number of patients Cycle 2.

1. Insomnia present in last 12 months

- Yes: 28 in Cycle 1, 30 in Cycle 2.

- No: 20 in Cycle 1, 18 in Cycle 2.

2. Sleep hygiene discussed

- No: 18 in Cycle 1, 18 in Cycle 2.

- Yes: 10 in Cycle 1, 15 in Cycle 2.

- N/A: 20 in Cycle 1, 15 in Cycle 2.

3. C B T i education given

- No: 25 in Cycle 1, 22 in Cycle 2.

- N/A: 20 in Cycle 1, 18 in Cycle 2.

- Yes: 3 in Cycle 1, 8 in Cycle 2.

4. Sleep reviewed

- Yes: 45 in Cycle 1, 43 in Cycle 2.

- No: 3 in Cycle 1, 5 in Cycle 2.

5. Name of hypnotic prescribed

- None: 31 in Cycle 1, 35 in Cycle 2.

- Zopiclone: 4 in Cycle 1, 2 in Cycle 2.

- Promethazine: 3 in Cycle 1, 4 in Cycle 2.

- Clonazepam: 2 in Cycle 1, 0 in Cycle 2.

- Zopiclone and clonazepam combination: 2 in Cycle 1, 1 in Cycle 2.

- Zopiclone and promethazine combination: 2 in Cycle 1, 0 in Cycle 2.

- Zopiclone and melatonin combination: 1 in Cycle 1, 0 in Cycle 2.

- Melatonin: 1 in Cycle 1, 0 in Cycle 2.

- Diazepam and promethazine combination: 1 in Cycle 1, 0 in Cycle 2.

- Melatonin and promethazine combination: 1 in Cycle 1, 2 in Cycle 2.

- Zolpidem: 0 in Cycle 1, 1 in Cycle 2.

- Diazepam and zopiclone combination: 0 in Cycle 1, 1 in Cycle 2.

- Daridorexant: 0 in Cycle 1, 1 in Cycle 2.

- Diazepam: 0 in Cycle 1, 1 in Cycle 2.

6. Number of hypnotics prescribed

- 0: 31 in Cycle 1, 35 in Cycle 2.

- 1: 10 in Cycle 1, 9 in Cycle 2.

- 2: 7 in Cycle 1, 4 in Cycle 2.

- 3: 0 in Cycle 1, 0 in Cycle 2.

Navigate back to Image 1:.

### Image 2: Long description

A large circle at the top-left contains the text: Consider risks including but not limited to: occupational, driving, physical health and caring responsibilities.

The main flowchart begins at the top center with an oval labeled Clinical review. An arrow points down to a rectangle asking: Does the patient experience a lack of sleep, poor quality of sleep or both that affects their quality of life?

- A No diamond to the right ends that path.

- A Yes diamond below leads to a rectangle asking: Is the patient able to engage with C B T-lite principles?

From this decision point, two paths emerge:

1. The No path leads down to a rectangle: Discuss lifestyle factors and sleep hygiene including caffeine, alcohol, exercise, diet content and timing, early morning light exposure and relaxation.

2. The Yes path leads down to a rectangle: Offer C B T-i principles of sleep scheduling, cognitive restructuring and sleep hygiene.

Both paths converge at a bottom-center rectangle: Review sleep. Is there sufficient improvement?

- A Yes diamond to the left leads to a final rectangle: Continue with successful intervention.

- A No diamond to the right leads to a final rectangle: Consider hypnotic (first line z-drugs) plus or minus medical review plus or minus referral to sleep clinic.

Navigate back to Image 2:.

